# Bridging gaps in dementia care across southeastern Europe: Regional challenges, cross-border innovation, and implementation barriers

**DOI:** 10.1177/22799036251361380

**Published:** 2025-07-30

**Authors:** Carolin Kurz, Ilir Alimehmeti, Marina Boban, Smiljana Kostic, Osman Sinanovic, Osman Kučuk, Shima Mehrabian, Latchezar Traykov, Ninoslav Mimica, Gabriela Novotni, Lea Pfaeffel, Vildan Dogan, Janine Diehl-Schmid, Panagiotis Alexopoulos, Christian Grünhaus, Zvezdan Pirtošek, Alexander Kurz

**Affiliations:** 1 Department of Psychiatry and Psychotherapy, LMU University Hospital, LMU Munich, Germany; 2 Faculty of Medicine, Department of Family Medicine, University of Medicine, Tirana, Albania; 3 Department of Neurology, University Hospital Centre Zagreb, Croatia; 4 Department of Neurology, Military Medical Academy, Belgrade, Serbia; 5 Medical Faculty University of Tuzla, Bosnia and Herzegovina; 6 Center for Dementia Sarajevo, Bosnia and Herzegovina; 7 Department of Neurology, Medical University of Sofia, Bulgaria; 8 University Psychiatric Hospital Vrapče, School of Medicine, University of Zagreb, Croatia; 9 Department of Cognitive Neurology and Neurodegenerative Diseases, University Clinic of Neurology, Medical Faculty, Ss Cyrill and Methodius University, Skopje, North Macedonia; 10 Office of International Science Cooperation, Bavarian Research Alliance (BayFOR), Munich, Germany; 11 Department of Psychiatry and Psychotherapy, Centre for Cognitive Disorders, School of Medicine, Technical University of Munich, Germany; 12 kbo-Inn-Salzach-Klinikum, Clinical Center for Psychiatry, Psychotherapy, Psychosomatic Medicine, Geriatrics and Neurology, Wasserburg/Inn, Germany; 13 Mental Health Services, Patras University Hospital, School of Health Sciences, University of Patras, Greece; 14 Global Brain Health Institute, Trinity College Dublin, The University of Dublin, Ireland; 15 Center for Nonprofit Organizations and Social Entrepreneurship, Vienna University of Economics and Business, Austria; 16 Department of Neurology, Ljubljana University Medical Centre, Slovenia

**Keywords:** health care delivery, holistic health, patient-centered care, health information technology, accessibility of health services, carers, community health services, health policy, health equity, aging population

## Abstract

Dementia is a growing challenge in Southeastern and Western Europe, with aging trends projected to accelerate in the former region. The region is facing critical gaps in dementia care due to rural-urban disparities, workforce shortages, and limited access to specialized services. Widespread reliance on informal caregiving and underdeveloped diagnostic infrastructure delay early diagnosis and equitable access to the healthcare system. This perspective article presents sustainable, regionally tailored solutions and innovative strategies from a multinational dementia network, which are aimed at improving care outcomes through collaboration, capacity building, and digital innovation. Tailored workshops and multilingual platforms have raised awareness and encouraged innovative care approaches. Meanwhile, mobile memory teams in underserved areas have been shown to enhance caregiver support and patient outcomes. The Circle of Care Hub framework was developed to bridge coordination gaps and promote equitable, integrated dementia care by linking healthcare providers, social workers, and families. Digital tools have been piloted to enhance professional training, interdisciplinary collaboration, and informal carer support. Addressing barriers such as limited services and workforce shortages is essential for sustainable improvements. Future initiatives should prioritize scalable interventions, such as mobile teams, digital platforms with evaluation mechanisms, and hybrid care models, while investing in early diagnosis, dementia centers and region-specific prevention strategies informed by robust epidemiological data. Achieving sustainable dementia care requires a combination of digital innovation and community-based solutions.

## Background

**Figure 1.**
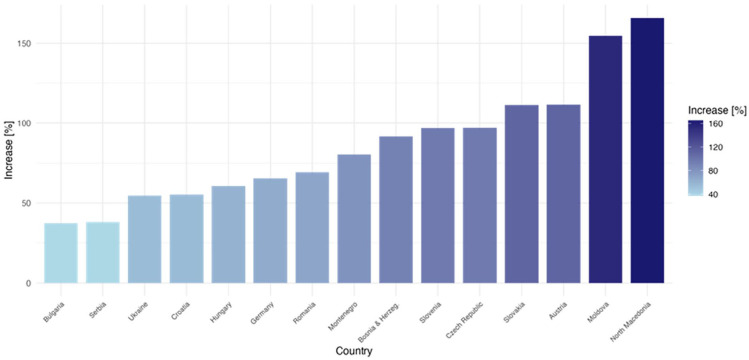
Increase in dementia prevalence (2019–2050). The graph shows the projected percentage increase in the population aged 65 and over in the countries of Southeastern and Western Europe up to 2050 and reflect the different demographic trends in the region, with accelerated ageing expected to have a greater impact in some countries than in others.

The rising prevalence of dementia is placing an ever-increasing burden on healthcare systems, challenging their ability to meet the complex needs of aging populations and adapt to the growing demand for specialized, multiprofessional care.^1,2^ This burden is expected to be particularly severe in Southeastern Europe, where rapid population ageing is projected to result in a faster increase in the proportion of older adults and persons living with dementia compared to Western Europe, putting additional pressure on already strained healthcare systems (see Figure 1 and Supplemental Table S1). ^3–5,65^

**Figure 2.**
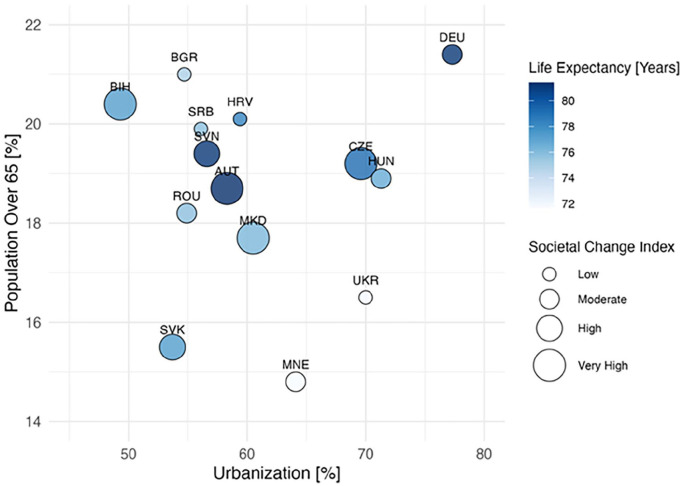
Demographic change and societal adjustment needs in European countries. A societal change index, calculated from the proportion of the population over 65, projected dementia increases, and life expectancy, highlights significant societal adaptation needs in countries with a very high index, such as Bosnia Herzegovina (BIH), North Macedonia (MKD) and Austria (AUT), while moderate to high index values are observed in Slovenia (SVN, Czech Republic (CZE), and Slovakia (SVK). In Bosnia-Herzegovina, Romania, Slovakia, Slovenia and Moldova, where up to 57.5% of the population lives in rural areas, these demographic constraints affect access to health services, social support and training of health workers.^5^

Southeastern Europe – as defined for this analysis- encompasses nine EU and five non-EU countries, thus being home to over 100 million people. This region shows substantial diversity in economic, demographic, and population characteristics. Life expectancy ranges from 71.8 years (Moldova) to 82.9 years (Austria), and population sizes vary from 82.7 million (Germany) to 0.6 million (Montenegro; Supplemental Table S2). Some countries have “young” populations with a low share of people aged 65 and older (e.g. Moldova, Montenegro), while others, such as Austria, Bulgaria, and Croatia, have aging populations surpassing the EU average.^6–9^ These demographic shifts are further complicated by rural-urban disparities, particularly in Bosnia-Herzegovina, Romania, and Moldova, where up to 57.5% of the population lives in rural areas, affecting access to health services, social support, and workforce development (see Figure 2, Supplemental Tables S2 and S3).

While research on dementia is advancing, important gaps remain in understanding regional prevalence patterns and care needs – highlighting the importance of further system-level investigation.^10–16^

Moreover, many healthcare systems lack standardized diagnostic tools and sufficient training for general practitioners.^13,15,16^ Expanding access to professional care, equipping caregivers with evidence-based interventions, and integrating person-centered therapeutic approaches are urgent priorities to better support persons living with dementia and their families.^17–21^

Reliance on informal care is still widespread, especially in rural areas.^12,22–30^ This dependency places substantial financial and emotional strain on caregivers, many of whom face inadequate formal support, mental health challenges, social isolation, and limited access to healthcare services.^31,32^ Additional barriers, including shrinking family sizes, the advanced age of caregivers, and low public awareness, contribute to delayed diagnoses and insufficient care.^24–26,33^ Furthermore, formal care options, such as institutional care, continues to be underdeveloped, with primary care providers often failing to diagnose dementia and underutilizing available treatments.^15,16,34–36^

Given dementia’s multifactorial etiology – including vascular, lifestyle, and environmental factors – comprehensive preventive strategies become increasingly relevant.^10,14,37^ Lifestyle modifications, comorbidity management, and early intervention offer significant opportunities to reduce dementia risk, especially since such preventive strategies are largely absent in the region.^10,11,14,37–42^

## Main text

To address these challenges, a multiprofessional network of dementia experts has been established to foster sustainable cross-border cooperation from Germany in the West to Bulgaria in the East and from the Czech Republic in the North to Albania in the South. The network focuses on promoting innovative interventions, including digital learning formats, caregiver support platforms and region-specific programs, aimed at improving prevention, diagnosis and long-term management of dementia. This article critically examines the ways in which these initiatives contribute to bridging the gaps and advancing equitable dementia care in the region.

### Collaborative initiatives addressing dementia care

This multinational consortium of medical practitioners from Southeastern European countries systematically studied the key challenges in dementia care across their countries and integrated results of a previous survey.^43^ The analysis highlighted critical areas for intervention, including rural-urban disparities, underdeveloped healthcare infrastructure, professional migration, and lack of specialized services. These findings were translated into regional initiatives and projects aimed at addressing gaps through interdisciplinary collaboration, digital innovations, and capacity building.

### Core projects

**Figure 3.**
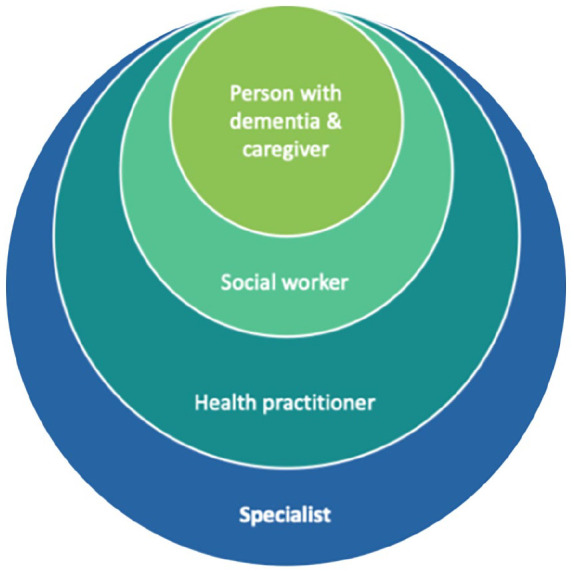
Circle of care hubs. The circle of care hub is a conceptual framework developed to demonstrate how dementia care could be improved through organized, multidisciplinary collaboration. At the heart of the model is the dementia coordinator, who connects healthcare providers, social workers, patient organizations and families to deliver coordinated, person-centered support. Although it is not a physical entity, the framework outlines mechanisms to bridge urban–rural divides, promote equitable distribution of resources, and foster dementia-friendly environments. By aligning local initiatives with transnational best practices, the model aims to reduce stigma, improve care outcomes and strengthen support systems, providing a vision for more inclusive and effective access to dementia care.

The Danubian Network for Dementia Education and Care (DANDEC), active from 2014 to 2018, established a collaborative network of 12 partner institutions and Alzheimer organizations across six countries, later expanding to 10. The project developed a regionally tailored e-learning program and addressed unmet needs in dementia care through multinational meetings. These meetings focused on improving professional skills, integrating evidence-based care frameworks, and developing assistive technology strategies. The project emphasized interdisciplinary collaboration through initiatives like the Dementia Academy and introduced the Circle of Care Hub framework to coordinate services among healthcare providers, social workers, and families under the guidance of dementia coordinators (see Figure 3).

The INDEED (Innovation for Dementia in the Danube Region) project, launched in 2018, further expanded cross-border collaborations among 11 countries. Its web-based platform offered multilingual, mobile-accessible content for public awareness, professional training, and business development. By addressing infrastructure limitations, INDEED promoted scalable interventions tailored to diverse contexts, including early diagnosis, multidisciplinary care, and dementia-friendly communities. The development of the “Interprofessional Management on Dementia” workbook and regional training sessions demonstrated the impact of strong organizational collaboration on improving care outcomes.

### Educational programs and digital solutions

A range of educational programs, including the Virtual Dementia Summer School and Dementia Master Classes, aimed to strengthen interdisciplinary dementia management through interactive sessions on person-centered care, treatment planning, and psychological support. The follow-up project STUDICODE (Stepping-Up Digital Competence in Dementia Education) improved the digital literacy of educators to develop online, flexible training modules for healthcare students and professionals. The modular structure, using interactive media such as videos and quizzes, ensured accessibility and relevance across different regions.

The ACCESS Dementia platform expanded these efforts by integrating features like an Easy Reading language version, simplified content, and sign language tools to improve accessibility for people with cognitive impairments, reading difficulties, or hearing loss. The platform’s community module connected users to organizations, dementia policies, and feedback mechanisms, while targeted workshops addressed the needs of 24-h caregivers. By combining digital flexibility with tailored content, ACCESS Dementia demonstrated the potential of hybrid solutions to address regional disparities and promote sustained learning.

### Socio-psychiatric intervention

The North Macedonia Interprofessional Dementia Care (NOMAD) project (2021–2024) introduced mobile memory teams, consisting of nurses and social workers, to provide diagnostic, therapeutic, and community-based support in rural areas.^22^ The project assessed the model’s feasibility through a cluster-randomized trial, demonstrating its effectiveness in improving access to care and strengthening national dementia strategies.^22^ However, challenges related to staffing shortages and financial constraints highlighted the need for scalable hybrid models combining mobile teams with telehealth services to optimize resource use.

## Key issues and future directions

Insights from the NOMAD project and related initiatives underline the need for broader analysis of care structures, diagnostic capacity, and implementation gaps to inform scalable strategies across the region.

### A critical analysis of care infrastructure and implementation gaps

Building on prior findings, critical infrastructure deficits—including limited community services and diagnostic tools—continue to delay diagnosis and increase caregiver burden. Targeted interventions must now focus on strengthening formal services, especially in underserved rural areas.^43–47^ These structural shortcomings are consistently reflected in regional research.^12,16,34^ Caregivers frequently report emotional strain, social isolation, and inadequate professional support, highlighting the urgent need to strengthen formal service networks.^29,31^ Tackling these deficits by expanding community-based services and mobile outreach is crucial to ensure sustainable and equitable dementia care infrastructure across the region, particularly in areas with shortages of specialists.^48–52^

### The importance of an early diagnosis

Even in high-income countries, dementia diagnoses are often delayed until the late stages of neurodegenerative diseases including Alzheimer’s disease.^53^ The extended preclinical phase offers a key opportunity for prevention, planning and shared decision-making, yet it remains widely underused.^3,54,55^

Scalable innovations, such as blood-based biomarkers (e.g. phosphorylated tau and glial fibrillary acidic protein), demonstrate strong concordance with cerebrospinal fluid biomarkers, amyloid and tau PET imaging, and clinical progression.^56–59^ This makes them promising tools for broader application, particularly when used alongside digital cognitive assessments or brief, validated questionnaires (e.g. the AD8, the IQCODE and the SCD-Q), as part of a stepwise diagnostic approach involving off-site screening and on-site confirmatory diagnostics, such as imaging or specialist evaluation.^60–62^ These markers could facilitate the early detection of disease and risk stratification, even in settings with limited resources. They promote the judicious use of advanced tools and improve early access to the health care system.^63,64^

### Prioritizing workforce development in dementia services

Primary care providers, especially general practitioners, are crucial in identifying persons with early cognitive decline and initiating preventive measures. Web-based training and continuing education programs help to close knowledge gaps and improve diagnostic work-up in both urban and rural settings, particularly because existing education and training programs of healthcare professionals remain disconnected from practical dementia care and lack standardized, evidence-based content.^18,20,43,44^ In order to address workforce shortages, efforts should focus on recruiting, training and retaining healthcare staff through targeted incentives and digital training platforms that enhance diagnostic capacity at the primary care level.^52^

### Highlighting differences in epidemiological trends

While some Western European countries report stabilizing or declining dementia incidence rates, which are likely linked to improvements in cardiovascular health, Southeastern Europe continues to experience an increase in prevalence driven by demographic aging, social disadvantage and a lack of adaptation in the health system.^3–5,65^ Furthermore, the lack of robust, large-scale epidemiological data from Southeastern Europe hinders targeted and effective policy responses.^10,11,13,14,25^ Furthermore, although the interplay between vascular, genetic and psychosocial risk factors is well documented in the literature, this is rarely reflected in regional preventive strategies.^37,40,66^ These findings underscore the urgent need for regionally tailored prevention and diagnostic strategies that systematically address modifiable lifestyle risk factors and promote health literacy.^55^

### Hybrid care models as a strategic opportunity

In light of the structural challenges – including limited diagnostic infrastructure, regional disparities, and the reliance on informal care – hybrid care models emerge not only as a necessary adaptation but as a strategic approach to improving equity, scalability, and system resilience in dementia care. Instead of updating outdated systems, Southeastern European countries can transition directly to tiered models, which have been proposed as optimal solutions by healthcare market experts.^67^ To have a sustained impact, such models must be embedded in robust evaluation frameworks and supported by cross-sector collaboration. Closing structural gaps and expanding access to targeted interventions is essential for achieving dementia care that is more equitable, cohesive and future-proof.

## Conclusion

Southeastern Europe is facing the dual challenge of an increasing prevalence of dementia and limited healthcare resources. However, these gaps offer an opportunity to reimagine healthcare delivery by adopting sustainable, equity-focused strategies.^55,68–70^ Digital innovation can help to bridge structural barriers by decentralizing information, supporting decision-making and improving knowledge transfer for patients, carers and providers.^44,71–74^ Yet disparities in digital infrastructure and low digital literacy still pose major obstacles.^75,76^

Achieving sustainable dementia care in Southeastern Europe will depend on effectively integrating digital innovation with locally adapted, community-based solutions, supported by cross-sector commitment and informed by robust data.

## Supplemental Material

sj-docx-1-phj-10.1177_22799036251361380 – Supplemental material for Bridging gaps in dementia care across southeastern Europe: Regional challenges, cross-border innovation, and implementation barriers Supplemental material, sj-docx-1-phj-10.1177_22799036251361380 for Bridging gaps in dementia care across southeastern Europe: Regional challenges, cross-border innovation, and implementation barriers by Carolin Kurz, Ilir Alimehmeti, Marina Boban, Smiljana Kostic, Osman Sinanovic, Osman Kučuk, Shima Mehrabian, Latchezar Traykov, Ninoslav Mimica, Gabriela Novotni, Lea Pfaeffel, Vildan Dogan, Janine Diehl-Schmid, Panagiotis Alexopoulos, Christian Grünhaus, Zvezdan Pirtošek and Alexander Kurz in Journal of Public Health Research

## References

[1] de Mendonça Lima CA Ivbijaro G . Mental health and wellbeing of older people: opportunities and challenges. Ment Health Fam Med 2013; 10: 125–127. 24427178 PMC3822658

[2] Organization WH . World Health Organization: Mental health of older adults. World Health Organization, 2022. https://www.who.int/news-room/fact-sheets/detail/mental-health-of-older-adults (2022).

[3] GBD 2019 Dementia Forecasting Collaborators. Estimation of the global prevalence of dementia in 2019 and forecasted prevalence in 2050: an analysis for the Global Burden of Disease Study 2019. Lancet Public Health 2022; 7: e105–e125. 10.1016/S2468-2667(21)00249-8 PMC8810394 34998485

[bibr4-22799036251361380] Commission E . The 2018 Ageing Report: Economic and Budgetary Projections for the EU Member States (2016-2070). European Commission, 2018.

[5] United Nations DoEaSA, Population Division. “World Urbanization Prospects Dataset”; PBL Netherlands Environmental Assessment Agency, “History Database of the Global Environment 3.3” [original data]. “World Urbanization Prospects Dataset”. 2023.

[bibr6-22799036251361380] PetrovaNN KhvostikovaDA . Prevalence, structure, and risk factors for mental disorders in older people. Adv Gerontol 2021; 11: 409–415.33993676

[bibr7-22799036251361380] Chirico I Chattat R Dostálová V , et al. The integration of psychosocial care into national dementia strategies across Europe: Evidence from the skills in Dementia Care (SiDECar) project. Int J Environ Res Public Health 2021; 18: 3422. 33806158 10.3390/ijerph18073422 PMC8036745

[bibr8-22799036251361380] European Commission. Directorate-general for employment SA and inclusion. Long-term care report – Trends, challenges and opportunities in an ageing society. Volume I. Publications Office, 2021.

[9] Eurostat. Population Demographic Indicators, https://ec.europa.eu/eurostat/databrowser/view/DEMO_GIND/default/table?lang=en (2023).

[10] Militaru M Lighezan DF Tudoran C , et al. Factors influencing the development and severity of cognitive decline in patients with chronic heart failure. Medicinar 2024; 60: 20241113. 10.3390/medicina60111859 PMC11596752 39597044

[bibr11-22799036251361380] Popovac A Mladenović I Krunić J , et al. Apolipoprotein ɛ4 allele and dental occlusion deficiency as risk factors for Alzheimer's disease. J Alzheimers Dis 2020; 74: 797–802. 32116259 10.3233/JAD-191283

[bibr12-22799036251361380] Lucijanić J Baždarić K Librenjak D , et al. A validation of the Croatian version of Zarit Burden interview and clinical predictors of caregiver burden in informal caregivers of patients with dementia: a cross-sectional study. Croat Med J 2020; 61: 527–537. 33410300 10.3325/cmj.2020.61.527 PMC7821365

[bibr13-22799036251361380] ÉrsekK KovácsT WimoA , et al. Costs of dementia in Hungary. J Nutr Health Aging 2010; 14: 633–639.20922339 10.1007/s12603-010-0309-1

[bibr14-22799036251361380] Yaneva-Sirakova T Traykov L . Mortality rate of high cardiovascular risk patients with mild cognitive impairment. Sci Rep 2022; 12: 11961. 35831445 10.1038/s41598-022-15823-1 PMC9279402

[bibr15-22799036251361380] Balogh R Imre N Papp E , et al. Dementia in Hungary: General practitioners' routines and perspectives regarding early recognition. Eur J Gen Pract 2020; 26: 7–13. 31601132 10.1080/13814788.2019.1673723 PMC7006793

[16] Heim S Busa C Pozsgai É , et al. Hungarian general practitioners' attitude and the role of education in dementia care. Prim Health Care Res Dev 2019; 20: e92. 10.1017/S1463423619000203 PMC6609992 32799975

[17] Vuic B Konjevod M Tudor L , et al. Tailoring the therapeutic interventions for behavioral and psychological symptoms of dementia. Expert Rev Neurother 2022; 22: 707–720. 35950234 10.1080/14737175.2022.2112668

[bibr18-22799036251361380] Ottoboni G Chirico I Povolná P , et al. Psychosocial care in dementia in European higher education: Evidence from the SiDECar ("Skills in DEmentia Care") project. Nurse Educ Today 2021; 103: 104977. 34051541 10.1016/j.nedt.2021.104977

[bibr19-22799036251361380] Pellegrino M Paoletti P Ortame L , et al. The Alzheimer's patients interaction through digital and arts (AIDA) program: A feasibility study to improve wellbeing in people with Alzheimer's disease. Prog Brain Res 2024; 287: 71–89. 39097359 10.1016/bs.pbr.2024.05.002

[bibr20-22799036251361380] Hvalič-Touzery S Skela-Savič B Macrae R , et al. The provision of accredited higher education on dementia in six European countries: an exploratory study. Nurse Educ Today 2018; 60: 161–169. 29132018 10.1016/j.nedt.2017.10.010

[21] Stojic J Petrosanec M Milosevic M , et al. The attitude and knowledge of medical students regarding dementia. Acta Neurol Belg 2022; 122: 625–630. 35429287 10.1007/s13760-022-01939-8

[22] Novotni G Taneska M Novotni A , et al. North Macedonia interprofessional dementia care (NOMAD) - personalized care plans for people with dementia and caregiver psychoeducation delivered at home by interprofessional teams. Front Dement 2024; 3: 1391471. 39081604 10.3389/frdem.2024.1391471 PMC11285573

[bibr23-22799036251361380] Bank W. World Development Indicators., https://data.worldbank.org/indicator (2024).

[bibr24-22799036251361380] Hanzevacki M Lucijanic J Librenjak D , et al. Unique contributions of specific neuropsychiatric symptoms to caregiver burden in informal caregivers family members of patients with dementia. Cogn Neuropsychiatry 2023; 28: 327–332. 37668258 10.1080/13546805.2023.2255338

[bibr25-22799036251361380] Lucijanić J Baždarić K Lucijanić M , et al. Predictors of health-related quality of life in informal caregivers of dementia patients in Zagreb, Croatia, a cross sectional study. Psychiatr Danub 2021; 33: 189–198. 35150485

[bibr26-22799036251361380] Sušac J Vukojević J Debogović S , et al. Share of and absolute costs of informal care in five subpopulations of outpatients with dementia in Croatia: a latent profile analysis. J Alzheimers Dis 2023; 94: 1417–1430. 37424466 10.3233/JAD-230161

[bibr27-22799036251361380] Jokinen N Gomiero T Watchman K , et al. Perspectives on family caregiving of people aging with intellectual disability affected by dementia: commentary from the International Summit on Intellectual Disability and Dementia. J Gerontol Soc Work 2018; 61: 411–431. 29583104 10.1080/01634372.2018.1454563

[bibr28-22799036251361380] Holmerová I Hort J Rusina R , et al. Costs of dementia in the Czech Republic. Eur J Health Econ 2017; 18: 979–986. 27785577 10.1007/s10198-016-0842-x

[bibr29-22799036251361380] Marhánková JH Honelová M . Making Sense of dementia: older adults' Subjective Representations of Dementia and Alzheimer's Disease. J Gerontol B Psychol Sci Soc Sci 2024; 79: 1–9. DOI: doi: 10.1093/geronb/gbae056 10.1093/geronb/gbae056 PMC11075728 38572717

[30] Nemcikova M Katreniakova Z Nagyova I . Social support, positive caregiving experience, and caregiver burden in informal caregivers of older adults with dementia. Front Public Health 2023; 11: 1104250. 36761127 10.3389/fpubh.2023.1104250 PMC9905841

[31] Woods B Arosio F Diaz A , et al. Timely diagnosis of dementia? Family carers' experiences in 5 European countries. Int J Geriatr Psychiatry 2019; 34: 114–121. 30246266 10.1002/gps.4997 PMC6586062

[32] Volpe U Amin H Ayinde OO , et al. Pathways to care for people with dementia: an international multicentre study. Int J Geriatr Psychiatry 2020; 35: 163–173. 31657091 10.1002/gps.5223

[33] Ostojić D Vidović D Baceković A , et al. Prevalence of anxiety and depression in caregivers of Alzeheimer's dementia patients. Acta Clin Croat 2014; 53: 17–21. 24974662

[34] Tomasović S Sremec J Košćak J , et al. Epidemiological characteristics of dementia treatment in Croatia. Psychiatr Danub 2016; 28: 170–175. 27287792

[bibr35-22799036251361380] Wagner EH . Chronic disease management: What will it take to improve care for chronic illness? Eff Clin Pract 1998; 1: 2–4. 10345255

[36] Maruta NA Yaroslavcev SA Kalenskaya GY , et al. Phenomenological analysis of suicidal behavior in patients with cognitive impairment in recurrent depressive disorder. Wiad Lek 2022; 75: 293–299. 35182138

[37] Bekić S Babič F Filipčić I , et al. Clustering of mental and physical comorbidity and the risk of frailty in patients aged 60 years or more in primary care. Med Sci Monit 2019; 25: 6820–6835. 31507272 10.12659/MSM.915063 PMC6753844

[bibr38-22799036251361380] Ifteni P Grudnikoff E Koppel J , et al. Haloperidol and sudden cardiac death in dementia: autopsy findings in psychiatric inpatients. Int J Geriatr Psychiatry 2015; 30: 1224–1229. 25790441 10.1002/gps.4277

[bibr39-22799036251361380] Janoutová J Kovalová M Machaczka O , et al. Risk factors for Alzheimer's disease: an Epidemiological Study. Curr Alzheimer Res 2021; 18: 372–379. 34420505 10.2174/1567205018666210820124135

[bibr40-22799036251361380] Kopchak OO Bachinskaya NY Pulyk OR . Vascular risk factors and cognitive functions in the patients with cerebrovascular disease. Wiad Lek 2020; 73: 2250–2254. 33310958

[bibr41-22799036251361380] Macesic M Lalic NM Kostic VS , et al. Impaired insulin sensitivity and secretion in patients with Alzheimer's disease: the relationship with other atherosclerosis risk factors. Curr Vasc Pharmacol 2017; 15: 158–166. 27599805 10.2174/1570161114666160905170644

[42] Pal T Iantovics LB Preg Z , et al. Risk factors for cognitive dysfunction amongst patients with cardiovascular diseases. Front Public Health 2024; 12: 1385089–20240913. 10.3389/fpubh.2024.1385089 PMC11427290 39346594

[43] Mehrabian S Schwarzkopf L Auer S , et al. Dementia care in the Danube region. A multi-national expert survey. Neuropsychiatr Dis Treat 2019; 15: 2503–2511. 31507321 10.2147/NDT.S161615 PMC6719840

[bibr44-22799036251361380] Alexopoulos P Novotni A Novotni G , et al. Old age mental health services in southern Balkans: features, geospatial distribution, current needs, and future perspectives. Eur Psychiatry 2020; 63: e88. 10.1192/j.eurpsy.2020.85 PMC7576530 32921324

[bibr45-22799036251361380] Europe M . An EU Action Plan for Better Cardiovascular Health, https://www.medtecheurope.org/wp-content/uploads/2021/05/an-eu-action-plan-for-better-cardiovascular-health_25_5_2021.pdf (2021).

[bibr46-22799036251361380] Koch M Fitzpatrick AL Rapp SR , et al. Alcohol consumption and risk of dementia and cognitive decline among older adults with or without mild cognitive impairment. JAMA Netw Open 2019; 2: e1910319. 10.1001/jamanetworkopen.2019.10319 PMC6777245 31560382

[47] Yaffe K Bahorik AL Hoang TD , et al. Cardiovascular risk factors and accelerated cognitive decline in midlife: the CARDIA Study. Neurol 2020; 95: e839–e846. 10.1212/WNL.0000000000010078 PMC7605504 32669394

[48] Organization WH . Global action plan on the public health response to dementia 2017–2025, https://www.who.int/publications/i/item/global-action-plan-on-the-public-health-response-to-dementia-2017—2025 (2017).

[bibr49-22799036251361380] Bundesministerium für Familie S, Frauen & Bundesministerium für Gesundheit. 2020, https://www.nationale-demenzstrategie.de https://www.nationale-demenzstrategie.de/fileadmin/nds/pdf/2020-07-01_Nationale_Demenzsstrategie.pdf.

[bibr50-22799036251361380] Lin SY . 'Dementia-friendly communities' and being dementia friendly in healthcare settings. Curr Opin Psychiatry 2017; 30: 145–150. 27997454 10.1097/YCO.0000000000000304 PMC5287032

[bibr51-22799036251361380] Maki Y Takao M Hattori H , et al. Promoting dementia-friendly communities to improve the well-being of individuals with and without dementia. Geriatr Gerontol Int 2020; 20: 511–519. 32207230 10.1111/ggi.13896

[52] Shannon K Bail K Neville S . Dementia-friendly community initiatives: an integrative review. J Clin Nurs 2019; 28: 2035–2045. 30554458 10.1111/jocn.14746

[53] Mate KE Magin PJ Brodaty H , et al. An evaluation of the additional benefit of population screening for dementia beyond a passive case-finding approach. Int J Geriatr Psychiatry 2017; 32: 316–323. 26988976 10.1002/gps.4466

[54] Jia J Ning Y Chen M , et al. Biomarker changes during 20 years preceding Alzheimer's Disease. N Engl J Med 2024; 390: 712–722. 38381674 10.1056/NEJMoa2310168

[55] Livingston G Huntley J Liu KY , et al. Dementia prevention, intervention, and care: 2024 report of the Lancet standing Commission. Lancet 2024; 404: 572–628. 39096926 10.1016/S0140-6736(24)01296-0

[56] Janelidze S Mattsson N Palmqvist S , et al. Plasma P-tau181 in Alzheimer's disease: relationship to other biomarkers, differential diagnosis, neuropathology and longitudinal progression to Alzheimer's dementia. Nat Med 2020; 26: 379–386. 32123385 10.1038/s41591-020-0755-1

[bibr57-22799036251361380] Palmqvist S Tideman P Mattsson-Carlgren N , et al. Blood biomarkers to detect Alzheimer disease in primary care and secondary care. JAMA 2024; 332: 1245–1257. 39068545 10.1001/jama.2024.13855 PMC11284636

[bibr58-22799036251361380] Wang X Shi Z Qiu Y , et al. Peripheral GFAP and NfL as early biomarkers for dementia: longitudinal insights from the UK Biobank. BMC Med 2024; 22: 192–20240513. 38735950 10.1186/s12916-024-03418-8 PMC11089788

[59] Qu Y Ma YH Huang YY , et al. Blood biomarkers for the diagnosis of amnestic mild cognitive impairment and Alzheimer's disease: a systematic review and meta-analysis. Neurosci Biobehav Rev 2021; 128: 479–486. 34245759 10.1016/j.neubiorev.2021.07.007

[60] Jorm AF . The informant questionnaire on cognitive decline in the elderly (IQCODE): a review. Int Psychogeriatr 2004; 16: 275–293. 15559753 10.1017/s1041610204000390

[bibr61-22799036251361380] Galvin JE Roe CM Powlishta KK , et al. The AD8: a brief informant interview to detect dementia. Neurol 2005; 65: 559–564. 10.1212/01.wnl.0000172958.95282.2a 16116116

[62] Tegethoff P Perneczky R Hufnagel A , et al. Development of a short version of the German subjective cognitive decline questionnaire (SCD-Q17): a principal component analysis approach to item reduction. Curr Psychol 2024; 43: 31056–31067.

[63] Owens DK Davidson KW Krist AH , et al. Screening for cognitive impairment in older adults: US Preventive Services Task Force Recommendation Statement. JAMA 2020; 323: 757–763. 32096858 10.1001/jama.2020.0435

[64] Canadian Task Force on Preventive Health C, Pottie K Rahal R , et al. Recommendations on screening for cognitive impairment in older adults. CMAJ 2016; 188: 37–46. 26622001 10.1503/cmaj.141165 PMC4695353

[65] Avan A Aamodt AH Selbaek G , et al. Decreasing incidence of stroke, ischaemic heart disease and dementia in Norway, 1990-2019, a Global Burden of Disease study: an opportunity. Eur J Neurol 2023; 30: 2267–2277. 37154405 10.1111/ene.15836

[66] Javor J Ďurmanová V Párnická Z , et al. Association of CD33 rs3865444:C˃A polymorphism with a reduced risk of late-onset Alzheimer's disease in Slovaks is limited to subjects carrying the APOE ε4 allele. Int J Immunogenet 2020; 47: 397–405. 32333488 10.1111/iji.12489

[67] Rajan DR Winkelmann J Kringos D , et al. Implementing the Primary Health Care approach: a primer. https://extranet.who.int/uhcpartnership/sites/default/files/reports/PHC%20primer.pdf?utm_source=chatgpt.com (2024). 41325501

[68] Lethin C Leino-Kilpi H Roe B , et al. Formal support for informal caregivers to older persons with dementia through the course of the disease: an exploratory, cross-sectional study. BMC Geriatr 2016; 16: 32. 26832354 10.1186/s12877-016-0210-9 PMC4734848

[bibr69-22799036251361380] McCabe M You E Tatangelo G . Hearing their voice: a systematic review of dementia family caregivers' needs. Gerontologist 2016; 56: e70–e88. 10.1093/geront/gnw078 27102056

[70] Albanese E Liu Z Acosta D , et al. Equity in the delivery of community healthcare to older people: findings from 10/66 Dementia Research Group cross-sectional surveys in Latin America, China, India and Nigeria. BMC Health Serv Res 2011; 11: 153–20110628. 21711546 10.1186/1472-6963-11-153 PMC3146820

[71] Anderson TJ Saman DM Lipsky MS , et al. A cross-sectional study on health differences between rural and non-rural U.S. Counties using the County Health Rankings. BMC Health Serv Res 2015; 15: 441. 26423746 10.1186/s12913-015-1053-3 PMC4590732

[bibr72-22799036251361380] Schmachtenberg T Monsees J Thyrian JR . Structures for the care of people with dementia: a European comparison. BMC Health Serv Res 2022; 22: 1372. 36401262 10.1186/s12913-022-08715-7 PMC9673874

[bibr73-22799036251361380] Ferri CP Jacob KS . Dementia in low-income and middle-income countries: different realities mandate tailored solutions. PLoS Med 2017; 14: e1002271. 10.1371/journal.pmed.1002271 PMC5370095 28350797

[74] Europe A . European Dementia Monitor 2023: Benchmarking dementia policies across Europe. https://www.alzheimer-europe.org/sites/default/files/2023-11/european_dementia_monitor_2023_final_0.pdf (2023).

[75] Woods L Eden R Macklin S , et al. Strengthening rural healthcare outcomes through digital health: qualitative multi-site case study. BMC Health Serv Res 2024; 24: 1096. 39300435 10.1186/s12913-024-11402-4 PMC11411842

[76] Erku D Khatri R Endalamaw A , et al. Digital health interventions to improve access to and quality of primary health care services: a scoping review. Int J Environ Res Public Health 2023; 20: 20230928. 10.3390/ijerph20196854 PMC10572344 37835125

