# Supplementary material for: Bridging gaps in dementia care across southeastern Europe: Regional challenges, cross-border innovation, and implementation barriers
Source: J Public Health Res. 2025 Jul 30;14(3):22799036251361380. doi: 10.1177/22799036251361380 (PMC12317160; doi:10.1177/22799036251361380)
Supplement: sj-docx-1-phj-10.1177_22799036251361380 – Supplemental material for Bridging gaps in dementia care across southeastern Europe: Regional challenges, cross-border innovation, and implementation barriers [file sj-docx-1-phj-10.1177_22799036251361380.docx]

**Supplementary Material: Tables for “Bridging Gaps in Dementia Care Across Southeastern Europe: Regional Challenges, Cross-Border Innovation, and Implementation Barriers”**

Content in the Supplement:

• **Table S1:** Number of persons living with dementia 2019 and 2050 in Southeastern Europe (1) (2).

• **Table S2:** Key demographic factors relevant to dementia care in Southeastern Europe (3) (4) (5) (6).

**Table S3.** Key economic factors influencing dementia care in Southeastern Europe (3) (4) (7).

**Table S1:** Number of persons living with dementia 2019 and 2050 in Southeastern Europe (1) (2).

| Country | persons living with dementia  2019 | persons living with dementia  2050 | Increase  [n] | Increase [%] |
| --- | --- | --- | --- | --- |
| Austria | 146.399 | 309.629 | 294.990 | 111 |
| Bosnia & Herzeg. | 48.081 | 92.167 | 44.086 | 92 |
| Bulgaria | 135.285 | 185.719 | 50.434 | 37 |
| Croatia | 83.429 | 129.479 | 46.050 | 55 |
| Czechia | 192.748 | 379.743 | 186.995 | 97 |
| Germany | 1.691.221 | 2.796.783 | 1.105.562 | 65 |
| Hungary | 183.870 | 295.379 | 111.509 | 61 |
| Moldova | 46.924 | 119.453 | 72.529 | 155 |
| Montenegro | 8.247 | 14.870 | 6.623 | 80 |
| North Macedonia | 28.279 | 75.147 | 46.868 | 166 |
| Romania | 341.195 | 577.177 | 235.982 | 69 |
| Serbia | 129.117 | 178.262 | 49.145 | 38 |
| Slovakia | 77.185 | 163.037 | 85.852 | 111 |
| Slovenia | 43.038 | 84.725 | 41.687 | 97 |
| Ukraine | 651.773 | 1.007.499 | 355.726 | 55 |
| EU 28 | 8.885.101 | 16.276.070 | 7.390.969 | 83 |

**Legend Table S1:** Several countries, including Moldova (155%), Northern Macedonia (166%), Slovakia (111%) and Austria (111%), are projected to experience a significant increase in the prevalence of dementia by 2050, along with significant population ageing, which will challenge health and social services.

**Table S2**: Key demographic factors relevant to dementia care in Southeastern Europe (3) (4) (5) (6).

| Country | Population [Mio.] (2022) | Life expectancy [years]  (2022) | Proportion of population 65+ [%] | | | | | | | | Urbanization [%]  (2022) | |
| --- | --- | --- | --- | --- | --- | --- | --- | --- | --- | --- | --- | --- |
|  |  |  | 2022 | | | 2030 | | | 2050 |  | | |
| Austria | 9.04 | 82,9 | 29,4 | | | 35,9 | | | 46,4 | 59,9 | | |
| Bosnia-Herzegovina | 3.23 | 78,2 | 18,3 | | | 22,2 | | | 28,8 | 50,6 | | |
| Bulgaria | 6.47 | 76,1 | 34,1 | | | 36,6 | | | 53,4 | 76,0 | | |
| Croatia | 3.89 | 79,7 | 35,6 | | | 41,5 | | | 51,8 | 57,6 | | |
| Czechia | 10.67 | 80,1 | 32,6 | | | 33,8 | | | 47,2 | 74,2 | | |
| Germany | 83.80 | 82,5 | 34,7 | | | 40,3 | | | 45,7 | 77,5 | | |
| Hungary | 9.64 | 77,4 | 31,7 | | | 32,6 | | | 45,5 | 72,3 | | |
| Moldova | 3.06 | 71,8 | 13,7 | | | 19,2 | | | 17,6 | 43,0 | | |
| Montenegro | 0.62 | 78,1 | 16,0 | | 19,4 | | | 24,7 | | | | 67,8 |
| North Macedonia | 2.06 | 78,3 | 17,1 | 18,4 | | | 26,7 | | | | 58,8 | |
| Romania | 0.19 | 77,5 | 30,3 | 42,0 | | | 50,2 | | | | 54,3 | |
| Serbia | 6.66 | 77,7 | 20,5 | 22,4 | | | 30,3 | | | | 56,7 | |
| Slovakia | 5.43 | 78,7 | 26,1 | 31,8 | | | 49,7 | | | | 53,8 | |
| Slovenia | 2.11 | 82,7 | 33,1 | 38,9 | | | 53,7 | | | | 55,1 | |
| Ukraine | 43.5 | 75,6 | 17,4 | 22,2 | | | 30,1 | | | | 68,8 | |

**Legend Table S2**: Bulgaria, Croatia and Germany have the highest percentages of the population aged 65 and over. In contrast, Moldova and North Macedonia have lower proportions of older people and lower levels of urbanization, highlighting disparities in ageing and urban-rural dynamics.

**Table S3:** Key economic factors influencing dementia care in Southeastern Europe (3) (4) (7).

| Country | Gross domestic product (GDP) per capita [US$, 2023] | Density of physicians  [per 10 000]  (2013-2020) | Nursing personnel  [per 10 000]  (2014-2020) | Health expenditure as a share of GDP  [%]  (2021) |
| --- | --- | --- | --- | --- |
| Austria | 37.430 | 53 | 102 | 12,1 |
| Bosnia and Herzegovina | 7.569 (2022) | 22 | 56 | 9,6 |
| Bulgaria | 7.850 | 42 | 47 | 8,6 |
| Croatia | 14.750 (2022) | 35 | 81 | 8,1 |
| Czechia | 18.370 | 42 | 92 | 9,5 |
| Germany | 35.590 | 44 | 124 | 12,9 |
| Hungary | 14.370 | 61 | 66 | 7,4 |
| Moldova | 5.714 (2022) | 31 | 61 | 7,8 |
| Montenegro | 6.650 (2022) | 27 | 57 | 11,6 |
| North Macedonia | 4.860 (2021) | 29 | 37 | 8,5 |
| Romania | 10.250 | 30 | 74 | 6,5 |
| Serbia | 4.820 | 31 | 72 | 10,1 |
| Slovakia | 14.590 | 36 | 79 | 7,8 |
| Slovenia | 18.550 | 33 | 105 | 9,5 |
| Ukraine | 4.534 | 30 | 67 | 8,0 |

**Legend Table S3**: In several countries (Bosnia and Herzegovina, Moldova, Montenegro, Ukraine) the number of health professionals, including physicians and nurses, is much lower than in other parts of Europe. (8)

### *References*

1. Collaborators GBDDF. Estimation of the global prevalence of dementia in 2019 and forecasted prevalence in 2050: an analysis for the Global Burden of Disease Study 2019. Lancet Public Health. 2022;7(2):e105-e25.

2. Europe A. Dementia in Europe Yearbook 2019 Estimating the prevalence of dementia in Europe 2019 [Available from: <https://www.alzheimer-europe.org/sites/default/files/alzheimer_europe_dementia_in_europe_yearbook_2019.pdf>.

3. United Nations WPPod. World Population Prospects (2022) – processed by Our World in Data. “Ages 65+” [dataset]. 2022.

4. Bank W. World Development Indicators. Washington, DC: World Bank; 2024 [Available from: <https://data.worldbank.org/indicator>.

5. Macedonia SSOotRoN. Population projections of the Republic of North Macedonia by 2070 2023 [Available from: <https://www.stat.gov.mk/publikacii/2023/Proekcii_2070_en.pdf>.

6. Eurostat. Population Demographic Indicators 2023 [Available from: <https://ec.europa.eu/eurostat/databrowser/view/DEMO_GIND/default/table?lang=en>.

7. Nations U. Sustainable Development Goals Data Portal. New York: United Nations; 2024 [Available from: <https://unstats.un.org/sdgs/dataportal>.

8. A. S, M.R. D-P. Migration of health workers: the WHO code of practice and the global economic crisis. Geneva: World Health Organization; 2014.
